# Meat Substitution with Oat Protein Can Improve Ground Beef Patty Characteristics

**DOI:** 10.3390/foods10123071

**Published:** 2021-12-10

**Authors:** Jase J. Ball, Ross P. Wyatt, Madison M. Coursen, Barry D. Lambert, Jason T. Sawyer

**Affiliations:** 1 Department of Animal Sciences, Tarleton State University, Stephenville, TX 76402, USA; jase.ball@zoetis.com (J.J.B.); blambert@tarleton.edu (B.D.L.); 2 Archer Daniels Midland, Decatur, IL 62526, USA; ross.wyatt@adm.com; 3 Department of Animal Sciences, Auburn University, Auburn, AL 36849, USA; mmc0067@auburn.edu

**Keywords:** cooked color, fresh color, ground beef patty, oat protein

## Abstract

The consumer acceptance of alternative plant-focused ingredients within the meat industry is growing globally. Oat protein is insoluble and used to increase product yield and fat retention. Furthermore, inclusion of oat protein can provide manufacturers another option for extending beef supplies. As the consumer diet shifts for improvements in nutritional density, oat protein is an alternative ingredient that lacks information on inclusion in a ground beef formulation. Coarse ground beef was allocated to one of four treatments, mixed with oat protein (0%, 1.5%, 3.5% and 4.5%), water, salt, pepper, textured vegetable protein, soy protein concentrate, and sodium tripolyphosphate. Meat blocks (*n* = 3 batches) were finely ground and formed into patties (N = 65/treatment). Patties were placed onto an expanded polystyrene tray, overwrapped with polyvinyl chloride film and displayed for 7 days. Instrumental color (L*, a*, and b*) decreased throughout simulated display (*p* = 0.0001). Increased usage rates of oat protein in patties resulted in greater cook yields (*p* = 0.0001). Objective measures of Allo-Kramer shear force values increased as oat protein inclusion rates increased (*p* = 0.0001). Oat protein can be incorporated in ground beef patties with positive effects on cook yield, but inclusion rate may have a deleterious impact on color and instrumental tenderness.

## 1. Introduction

Ingredient technologies considered for ground beef are growing in importance not only for meat manufacturers, but also consumers, as it becomes increasingly more expensive to purchase beef products [1]. Meat products remain a critical staple within consumers diets and their consumption can be influenced by sensory characteristics, environmental influencers linked to social, economical, political, or even geographical factors [2]. Consumers within the retail setting are becoming more conscious in their purchasing decisions with regard to price increases and ingredient labels. Therefore, an obvious need for providing a high quality, yet economical, healthy ground beef product is becoming increasingly important. The continued development and investigation of value-added technologies is necessary. Within global markets, or specifically in food service applications, ground beef has become an inexpensive alternative to steaks and roasts, as pricing of fresh meat can fluctuate from week to week [3]. Nonetheless, the consumption of ground beef or value-added product is the predominant source of beef intake domestically as confirmed through previous research efforts [3].

In an effort to achieve a more sustainable food production system, the investigation of alternative production practices is needed. To reach the benchmark “2030 Agenda for Sustainable Development” [4], meat and food industries need to create foods that are nutritionally superior, wholesome, and price conscious [5] based on consumer demands. Meat products have been linked to heart disease, diabetes, and cancer [6,7] and the creation of alternative meat focused options that contribute to human health could be advantageous to the meat industry and consumers globally. The use of plant-based proteins within the retail meat counter are gaining in popularity with growth expected to exceed 10% by 2035 [8]. In previous work by this lab, the investigation into pea, rice, and oat protein reported ground beef patty characteristics to be minimally altered [9]. Oat protein was initially selected from previous research efforts to further illicit additional information about meat-based products enriched with plant proteins in a ground beef patty.

It is estimated that the demand and marketing of fresh meat products will increase by 7.35% globally through 2025 [10]. As the consumer becomes more “flexible” in their dietary habits, the rise in global population, reduction of greenhouse gases, religious or ethical issues has provided meat and food industries an opportunity to investigate the use of plant-based proteins. The launch of plant-based meat products has transitioned from niche to mainstream with an estimated 6485 products globally since 2015 [11].

Furthermore, the necessity for new ingredient technologies within ground beef programs also lies in the simple economies of supply and demand. It is estimated the demand for beef has remained consistent as it is one of the few protein sources to remain palatable to the consumer [12] as a result of intramuscular fat deposition and less dependent on value-added creation. As the beef industry shifts to a more coordinated system capable of delivering more branded beef products into smaller niche markets, manufacturers are seeking methods for manufacturing to remain profitable and validate greater production [13]. Unfortunately, due to many unforeseen factors, the supply of beef has decreased dramatically over the past few years, causing beef prices to increase. The arrival of a global pandemic (COVID-19) led to countless disruptions throughout the meat industry with greater impacts occurring first within the food service industries [14]. Beef is unlike the other major protein sources such as chicken and pork where stable supply remains, the beef supply can be highly volatile, and dependent on factors such as rural development and climate changes [15]. Stable demand, decreasing supply, and rising prices foster favorable conditions for alternative techniques such as adding oat protein to maintain production volume [16].

Previously, added ingredients (oat flour, oat bran, soy, textured vegetable protein) within meat product formulations have been investigated that lead to altering cooking yields, reductions in fat intake, and can provide a source of functional foods to improve human body functions [17,18,19]. The inclusion of vegetable proteins originated within a meat formulation focused initially on improving cooking yield and product textures only. As consumer interest for an alternative diet grows in popularity, it is valuable information to capture the characteristic impacts and functionality [20] of including vegetable focused ingredients within a meat formulation. With an initial focus on value-added meat product formulation within a fast-cooking category [21], it is necessary to identify impacts to additional meat-based products enriched with oat proteins that ingredients may cause in patty quality such as texture, color and cooking yield.

Other factors that have altered the supply of beef can be environmentally focused (drought), and production capacity (labor shortages) throughout the United States (U.S.), often forcing cattle producers to reduce herd size [22]. Nonetheless, meat and meat products are a large influencer of human diets within developed countries. As meat consumers focus on securing a balanced diet, the creation of new meat products enriched with plant proteins can be challenging. Countless aspects of a meat formulation should be considered during the manufacturing phases to identify any alterations to the characteristics that influence consumer sensory and nutritional content [2]. Therefore, the objective of the current study was to identify formulation percentages of oat protein that could be integrated into a ground beef patty and the subsequent implications on fresh and cooked patty characteristics.

## 2. Materials and Methods

Coarse ground beef (81% lean) was purchased from a commercial beef processor (National Beef, Inc., Kansas City, MO, USA), transported to the Tarleton State University Meat Laboratory and stored at 2 °C. Coarse ground beef was allocated to one of four treatments containing 0, 1.5, 3.5 and 4.5 % oat protein in 4.91 kg batches (three batches/treatment). Oat protein was purchased from a commercial ingredient manufacturer (SunOpta Ingredients, Edina, MN, USA). All batches of coarse ground beef were formulated to contain water (17.0%), salt (0.75%), phosphate (0.30%), black pepper (0.25%), textured vegetable protein (5.0%, Archer Daniels Midland, Decatur, IL, USA), and soy protein concentrate (1.0%, Archer Daniels Midland, Decatur, IL, USA). Ground beef assigned to a positive control included the same ingredient formulation with the exclusion of oat protein (0%). Coarse ground beef along with added ingredients were mixed for 5 min in a butcher boy ribbon mixer (Model 250M, Selmer, TN, USA). Upon mixing, meat block formulation was ground once through a 3.175 mm diameter cm plate with a four-blade knife using a Biro mixer/grinder. Ground beef treatments were then formed into 227 g patties using an automated patty former (Hollymatic, Super 54, Model No. 65376, Hollymatic Corporation, Countryside, IL, USA) that were 1.9 cm thick and 15.24 cm in diameter. All patties to be analyzed were assigned randomly from each batch (*n* = 3 batches) for Allo-Kramer shear force, cooking time, cooking yield, moisture loss, and cooked color were individually vacuum packaged (Multivac C500, Kansas City, MO, USA) in a 3 mil vacuum barrier package; and frozen at −10 °C for 30 days until laboratory analysis could be completed. Patties allocated from each batch (*n* = 3 batches) to fresh instrumental surface color and lipid oxidation were placed onto a Styrofoam tray, wrapped in polyvinyl chloride film (PVC), and displayed for 7 days in a three-tiered (Model 60DXB-N Turbo Air Inc., Long Beach, CA, USA) refrigerated display case operating at 4 °C with four 15 min defrost cycles every 24 h. The lighting intensity of each shelf was 2300 lux (ILT10X, International Light Technologies, Peabody, MA, USA), and storage temperatures were monitored using a data recorder (TD2F, Thermoworks, American Fork, UT, USA) placed in the center of each shelf. Fresh patty packages were rotated daily throughout the shelves from side to side and front to back to eliminate temperature variation and to simulate consumer package shifting at the retail counter.

### 2.1. Cooking Methods

Prior to analyzing patties for Allo-Kramer shear force, cooking yield, and instrumental cooked color, frozen patties were thawed for 12 h at 4 °C. Thawed patties were cooked on an electric griddle (Model 07062, Presto, Eau Clair, WI, USA) pre-heated to 176 °C and flipped over every 2 min throughout the cooking process until the internal temperature in the geometric center of the patty reached 71 °C. Internal temperatures of the patties were monitored during cooking with a digital thermometer (Model CD28K Type, Comark Instruments, Beaverton, OR, USA). After cooking, patties were allowed to cool to room temperature 23 °C for no more than 30 min.

### 2.2. Allo-Kramer Shear Force

Allo-Kramer shear force was performed using an Instron Universal Testing Machine (Model 1011, Instron Corporation, Norwood, MA, USA). A standardized specimen size (2 × 5 cm²) was cut, and placed flat in a 5-blade Allo-Kramer shear cell attached to a 500 kg load cell with a standard load range setting of 100 kg and a crosshead speed of 500 nm/min. Kilograms of shear force was then converted to Newtons of force: (kg of shear force × 9.8).

### 2.3. Cooking Time and Cooking Yield

Prior to cooking, each patty was removed from their respective package, blotted dry with a paper towel and weighed on a balance. Patties were then cooked using the procedures described above. After cooking, patties were cooled to room temperature (23 °C) for no more than 30 min prior to re-weighing each patty and recording post cooking weight. Cooking yield was calculated using following formula: [cooked weight/raw weight] × 100.

### 2.4. Instrumental Cooked Color

After cooling for 5 min, cooked patties were sliced through the geometric center using a Hobart slicer (Model 3813, Hobart, Troy, OH, USA). Immediately after slicing, instrumental cooked color readings were collected by scanning the internal side of each patty using a HunterLab MiniScan XE Plus (Model 45/0-L, Hunter Associates Laboratory Inc., Rustin, VA, USA). Color readings (L*, a*, b*) were recorded with an Illuminant A light source, 10° standard observer, and a 250 mm viewing aperture using the Commisision Internatonale de l’ Eclairge (CIE L*a*b*) color scale [23]. At random locations on the patty, three readings were measured for lightness (higher “CIE L*” value is indicative of a lighter color), redness (higher “CIE a*” value is indicative of a redder color), and yellowness (higher “CIE b*” value is indicative of a more yellow color). Instrumental cooked color values were used to calculate hue angle (representing a change from the true red axis) as: tan^−1^(CIE b*/CIE a*), chroma (representing the total color) as: (CIE a*² + CIE b*²)^1/2^; and red to brown were calculated using the reflectance ratio of 630 nm/580 nm [24].

### 2.5. Instrumental Fresh Color

Fresh instrumental color readings were measured on fresh, never-frozen patties (*n* = 20/treatment) throughout a simulated retail display period on d 0, 3, 5, and 7 at 17:00. Each day, the colorimeter was calibrated prior to data collection using the standard black and white tiles [25]. Each patty (*n* = 10) was scanned three times to determine an average surface color reading for L*, a*, and b* values (Illuminant A) using a HunterLab MiniScan XE Plus (Model 45/0-L, Hunter Associates Laboratory Inc., Rustin, VA, USA). Instrumental color values were used to calculate hue angle (representing a change from the true red axis) as: tan^−1^(b*/a*), chroma (representing the total color) as: (a*² + b*²)$\frac{1}{2}$, and oxymyoglobin was calculated to measure the reflectance ratio of 630 nm/580 nm [24].

### 2.6. Lipid Oxidation

Fresh patties from the simulated retail display cases were used for testing lipid oxidation (*n* = 10/day/treatment) and removed from the display case on d 0, 3, and 7, vacuum packaged and frozen until further analysis. Prior to analysis, frozen patties were thawed for 12 h at 4 °C. After thawing, 2 g from each patty was homogenized (AHS 250, VMR Power Max, Radnor, PA, USA) with 8 mL of cold (1 °C) 50 mM phosphate buffer mix standardized to a pH of 7 and containing 0.1% EDTA, 0.1% n-propyl gallate, and 2 mL trichloroacetic acid (Sigma, St. Louis, MO, USA). Samples were then filtered through Whatman No. 4 filter paper, and duplicate 2 mL aliquots of clear supernatant were then transferred into 10 mL borosilicate tubes, mixed with 2 mL of 0.02 M 2-thiobarbituric acid reagent (Sigma, St. Louis, MO, USA), and boiled at 100 °C for 15 min. Immediately after boiling, tubes were placed into an ice bath for 15 min. Absorbance was measured at 533 nm with a spectrophotometer (Thermo Fisher Scientific, model Genesys 10 UV, Waltham, MA, USA) and multiplied by a factor of 12.21 to obtain the TBARS (mg malonaldehyde/kg of meat) value [26].

### 2.7. Moisture Loss

Fresh patties from the simulated retail display cases were measured for percent moisture analysis using the patty assigned for lipid oxidation (*n* = 10/day/treatment) On days 0, 3, or 7, fresh, never-frozen patties were removed from the retail display case, and patties were re-weighed on a balance prior to vacuum packaging and freezing until lipid oxidation analysis could be completed. Moisture loss was determined using the following formula: {[Initial weight − Final weight] ÷ [Initial weight] × 100}.

### 2.8. Experimental Design and Statistical Analysis

Data were analyzed using the Mixed procedures of SAS (SAS Institute, Inc., Cary, NC, USA). All data were analyzed as a randomized complete block design with beef patty serving as the experimental unit and blocked by batches within treatment. Analysis of variance were generated with treatment as the lone fixed effect, and block as the lone random effect, while patty replication was used as a repeated measure for moisture loss, instrumental color, cook yield, and instrumental tenderness. Least squares means were generated, and, when significant (*p* ≤ 0.05) F-values were observed, least squares means were separated using pair-wise *t*-test (PDIFF option).

## 3. Results and Discussion

### 3.1. Allo-Kramer Shear Force

Allo-Kramer shear force values increased (*p* < 0.0001) as the percentage of formulated oat protein usage increased within the ground beef patty formulation (Table 1). Previous research on functional ingredients such as oat protein report textural parameters may decline as a result of protein coagulation [27]. Nonetheless, texture may not be the driver of ground beef patties formulated with oat protein. Consumers today are seeking healthier, leaner options when purchasing beef products as the National Consumer Retail Beef Study and the National Beef Market Survey [28,29] indicated consumers would purchase more beef if the fat were closely trimmed or contained less fat. The increase in objective measured texture suggests that the inclusion of oat protein within a ground beef formulation should be considered and evaluated further in an effort to avoid negative impacts on objective tenderness of the patty. While the objective focus of the current study was on physical characteristics of the patties, it would be advantageous to conduct sensory evaluation to confirm objective measurements reported in the current study.

### 3.2. Cooking Time

Cook times were greater (*p* < 0.0001) for ground beef patties containing oat protein than patties containing no (0%) added oat protein (Table 1). The addition of oat protein in ground beef patties required more time on the grill for patties to reach an internal temperature of 71 °C. It has been reported [30] that variation in fat content within a beef patty can also alter cooking time. Fat content of patties comprised of less fat and more lean require greater cooking times [31]. The current results tend to agree with previous results on cooking times being altered as fat content or more specifically formulation of the ground beef patty is modified. It is possible that this extension of cooking time within the quick-service restaurant setting could alter food service application practices and preparation itineraries.

### 3.3. Cooking Yield

Cooking yield is considered an important trait measured during this study as the possible outcome for value-added patties is a fully cooked, ready-to-heat customer application. Cooking yield results indicate that the cooking yield increased (*p* < 0.0001) as the percentage of oat protein increased (Table 1). The results within the current study agree with previous research [9,30] reporting cooking yields were improved with plant proteins or pea fiber in a ground beef application. The increase in cooking yield resulted in a greater percent of moisture retained during the cooking process and could result in less lean trim during product formulations in a prepared food facility. Previous research [31] reported that cooking losses occur during the cooking process because moisture and fat are lost as a result of heating. With the addition of oat protein, moisture retention increased, resulting in a possibility to extend the beef supply, by allowing more ground beef to be produced. Additional research conducted on ground beef has [32,33,34,35,36] reported that the impact of reducing fat content of beef patties can alter characteristics that consumers may deem important. In general, as the fat content is reduced from 25 to 30% downward to 5 to 10%, cooking loss, drip loss, juiciness, beef flavor, tenderness, oily mouth coating, and consumer demand decreases. Moreover, as fat content declines, myoglobin content, cooking times, beef patty hardness, cohesiveness, springiness, crumbliness, density, and Allo-Kramer shear force values increase [32,33,34,35,36]. The current results suggest that as fat content decreases or meat extender ingredients increase there could be negative consequences to sensory attributes of the ground beef patty. Nonetheless, additional research should be completed evaluating the sensory taste parameters that oat protein inclusion in ground beef patties may create.

The ability of the added oat protein to retain greater amounts of moisture throughout the cooking process suggests that the added oat protein may provide the consumer with a juicier ground beef patty without added fat. Although processed meats sausages emulsified or coarse and ground beef are typically greater in fat content than retail cuts, these products have the greatest opportunity for fat reduction along with water retention because their nutrient composition can potentially be altered by reformulation with a fat replacement or a combination of fat replacements [37].

### 3.4. Moisture Loss

There was no (*p* = 0.6796) interactive impact (treatment × day) for percent moisture loss throughout the 7 day retail display (Table 1). The main effect for percent moisture loss did not differ (*p* > 0.7496) among oat protein inclusion treatments during the simulated retail display period. However, moisture loss tended to be greater in patties containing 1.5% and 3.5% oat protein. As expected percent moisture loss was greater (*p* < 0.0002) on day 7 of simulated display (data not reported) for moisture losses. Moisture loss results during the simulated retail display differ from previous results [38] reporting that added oat protein to low-fat (<10%) beef patties can improve cooking yield, in addition to fat and moisture retention, when compared to 20% fat control patties. However, few if any studies exist measuring the fresh qualities of ground beef patties during simulated retail display settings containing plant-based ingredients. It is plausible that the limited impact on moisture loss percentages within the current study during the display period may be attributed to the water binding ability of oat protein.

### 3.5. Instrumental Fresh Color

Fresh ground beef patties allocated from all batches and packaged in aerobic packages were displayed in conditions to measure surface color variations for a simulated 7 day period. Moreover, the visual appearance of meat can be affected by several different factors. These factors of chilling rate, diet, breed, and housing have all been reported to have a profound influence on meat surface color during simulated display conditions [39,40,41]. There was no (*p* > 0.6301) interactive impact (treatment × day) on fresh surface color lightness (L*), or yellowness (b*) of the beef patties. Fresh instrumental surface color value (L*) main effects for ground beef patties enriched with oat protein were lighter and less yellow (*p* < 0.0001) when presented in retail display settings (Table 2), whereas the fresh surface color main effects of enriched patties throughout the display period suggest ground beef patties became darker and less yellow (*p* < 0.0001) through day 7 of the simulated display period (Table 3).

**Figure 1:**
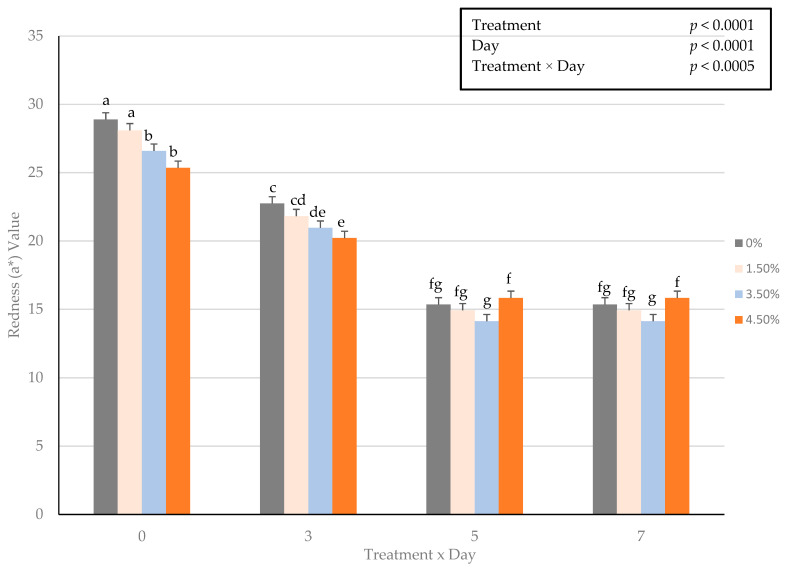
Interactive effect of treatment × day on fresh surface color redness (a*) values of ground beef patties during a simulated retail display. Bars lacking common letters differ (*p* < 0.05).

**Figure 2:**
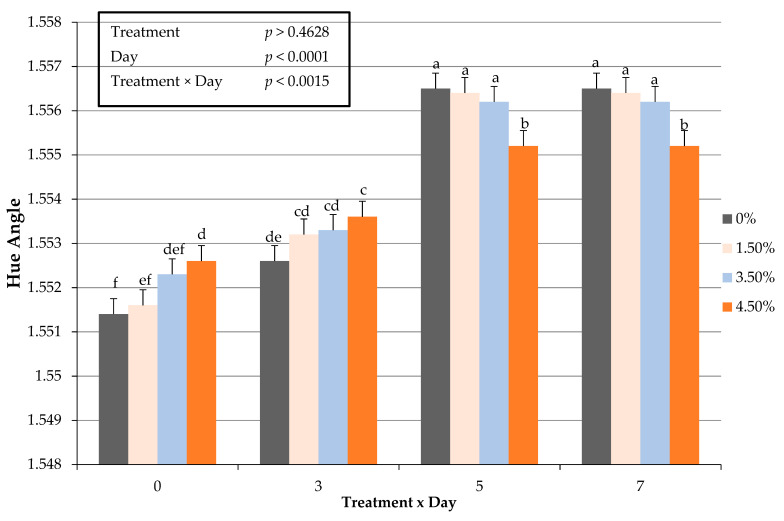
Interactive effect of treatment × day on fresh surface color hue angle values in ground beef patties during a simulated retail display. Bars lacking common letters differ (*p* < 0.05).

**Figure 3:**
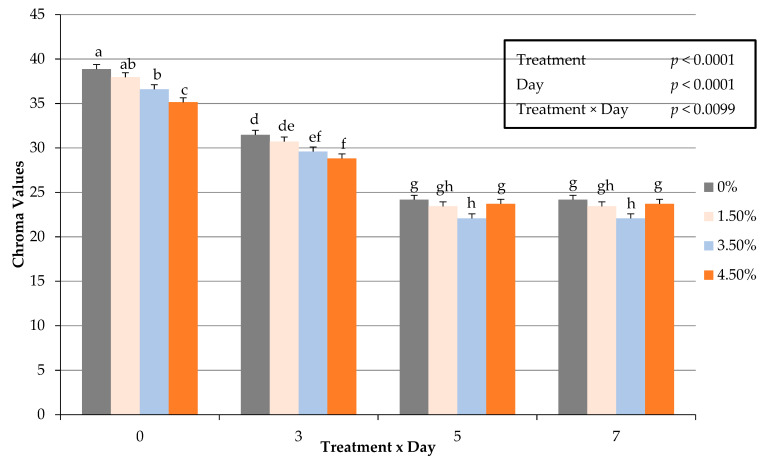
Interactive effect of treatment × day on fresh surface color chroma (vividness) values of ground beef patties during a simulated retail display. Bars lacking common letters differ (*p* < 0.05).

**Figure 4:**
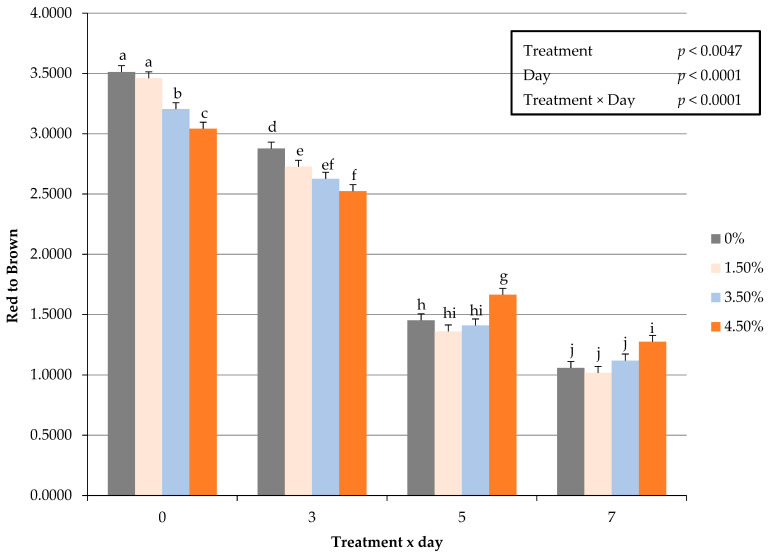
Interactive effect of treatment × day on fresh surface color values red to brown (630:580 nm) of ground beef patties during a simulated retail display. Bars lacking common letters differ (*p* < 0.05).

An interactive impact of treatment × day for fresh surface color redness (a*) values occurred (*p* < 0.0005) throughout the display period (Figure 1). These changes in fresh surface redness may be attributed to a decrease in the volume of ground beef used, and thus resulting in less myoglobin available for oxidation in a traditional overwrapped packaging platform. The decline in redness (a*) values throughout the display period agrees with previous research [9] by this lab on ground beef patties enriched with plant proteins. Redness values did not differ during fresh color display when using oat protein in a ground beef patty formulation [9]. In regard to fresh instrumental color yellowness (b*), surface color values decreased with increasing use of oat protein and could have hindered the fresh color development. Furthermore, the current study evaluated hue angles, as an expression of the change in color from the true red axis. The interactive influence of treatment × day on hue angles were greater (*p* < 0.0015) as the percentage of oat protein increased (Figure 2). The changes in hue angles suggest as the duration of simulated display increased patty redness moved further from the true red axis. These findings suggest that it is plausible that a threshold limit of oat protein within fresh ground beef patty may exist. Chroma is defined as the intensity or vividness of a surface color, but was altered with the inclusion of oat protein (Figure 3). The interactive effect (treatment × day) resulted in oat protein causing surface vividness changes during the 7 d display period (*p* > 0.0099) causing patties to become less vivid at the conclusion of the simulated retail display. During the simulated retail display, it is evident that increased formulations of oat protein (treatment × day) resulted in fresh surface color changes (*p* < 0.001) causing values of calculated spectral values red to brown (630:580 nm) to decline (Figure 4). The results on fresh surface color suggests that the enrichment of ground beef patties with oat protein may impart some influence on the surface color of fresh meat patties [27]. It is plausible that the combination of aerobic packaging materials (overwrapped) and the greater exposed surface area of ground beef reached the end of shelf life for surface color by day 5 of the simulated display. These external factors likely caused surface color variations to be limiting among the patties. Furthermore, the impact of these changes caused by oat protein should be investigated further to assess a deeper influence on visual sensory factors as measured by trained or consumer panelists.

### 3.6. Thiobarbaurtic Acid Reactive Substances (TBARS)

Fresh beef patties enriched with oat protein were stored in aerobic packaging materials during a simulated retail display period for 7 days and measured for lipid oxidation. There was no interactive impact (treatment × day; *p* = 0.2314) for oat protein and retail display day on TBAR values. Lipid oxidation main effect values (Table 4) did not differ from ground beef patty samples containing oat protein (*p* > 0.2995). Interestingly, TBARS were greater (*p* < 0.0001) initially on day 0, but declined throughout the simulated display period. The results from this study coincide with previous results [42] that the addition of legume flours to a meat formulation do not have a detrimental effect on lipid oxidation during retail display. Lipid oxidation is one of the main limiting factors for the quality and acceptability of meat and meat products [43] that has been used throughout the meat industry. The degradation of lipids with increasing inclusion rates of oat protein suggests that the use of oat protein within an industry setting for ground beef patties could be applicable. These results may enhance the marketability of an oat protein enriched ground beef patty in light of discernable differences in TBAR values. Furthermore, these results could reduce the number of markdowns and throwaways retail providers encounter weekly with regard to their meat counters.

### 3.7. Instrumental Cooked Color

Instrumental values for internal cooked color of ground beef patties were altered (*p* < 0.05) with the inclusion of oat protein (Table 5). Internal cooked color of the patty was lighter (L*) but did not differ (*p* = 0.9165) among all patty treatments after cooking (Table 4). As expected, cooking resulted in denaturation of myoglobin, but oat protein was not a contributing factor for changes of internal lightness (L*), whereas patties were less red (*p* < 0.0001), had greater hue angles (*p* < 0.0001), and a greater change (*p* < 0.0002) from red to brown after cooking. However, oat protein did not (*p* = 0.5507) impact yellowness values for cooked ground beef patties. Nonetheless, patties containing at least 3.5% oat protein were less (*p* < 0.0004) vivid after cooking. Although the differences were numerically minimal for cooked instrumental color of meat proteins containing oat protein, these results do not agree with [37], which evaluated the internal cooked color of ground beef patties with varying lean to fat ratios. The previous study [44] reported that there was not a difference in internal cooked color (*p* > 0.05) in ground beef patties. It is apparent that the variation in fat quantity could be a contributing factor for internal color fluctuations reported within the current study. The evaluation of previous research [44] along with the current results suggest that ground beef patties with different lean-to-fat ratios and the inclusion of oat protein may cause variation of the internal cooked color of the ground beef patty.

## 4. Conclusions

Results from this study suggest that oat protein could be utilized within a ground beef application. Furthermore, the inclusion of oat protein can provide ground beef patty manufacturers with clean label alternatives to use in a meat analogue system. Interestingly, the results from the current study suggest that oat protein could be utilized to increase cooking yield, but overuse could result in negative effects relative to objective tenderness measurements. However, additional efforts investigating sensory traits of taste and visual color are necessary to further illicit changes to a ground beef patty that may be detected by consumers. Nonetheless, the use of oat protein throughout the ground beef industry may be considered to off-set the reduction in beef supplies while meeting the demand for a cheaper, value-focused meat product in the retail or foodservice sectors.

## Figures and Tables

**Table 1:** Main effects for instrumental tenderness, cook yield, cook time, and moisture loss on ground beef patties containing oat protein.

	Treatment					
	0%	1.5%	3.5%	4.5%	SEM *	*p*-Value
Allo-Kramer Shear Foce, N ^1^	394.19 ^b^	410.42 ^b^	474.54 ^a^	496.99 ^a^	8.854	0.0001
Cook Yield, % ^2^	85.01 ^c^	88.87 ^b^	88.85 ^b^	90.47 ^a^	0.364	0.0001
Cooking Time, s ^3^	585.20 ^c^	593.80 ^c^	702.55 ^a^	652.55 ^b^	12.951	0.0001
Moisture Loss, % ^4^	0.5604	0.5905	0.5830	0.5598	0.167	0.7496

^1^ Allo-Kramer shear force values are measure of tenderness in kilograms of shear force and divided by the sample weight to determine the shear force in kg/g of sample. This value was then converted to Newtons of force by multiplying kg of shear force × 9.8. ^2^ Cook Yield = ((cooked patty weight/initial patty weight) × 100). ^3^ Cooking Time = time in seconds for the patty to reach an internal temperature of 71.1 °C. ^4^ Moisture Loss = {[Initial weight − Final weight] ÷ [Initial weight] × 100. ^a,b,c^ Means lacking a common superscript letter differ (*p* ≤ 0.05). * SEM, Standard Error of the Mean.

**Table 2:** Main effect of treatment on instrumental fresh surface color of ground beef patties during a simulated retail display.

	Treatment					
	0%	1.5%	3.5%	4.5%	SEM *	*p*-Value
Lightness (L*) ^1^	53.5732 ^c^	54.0109 ^b^	54.3325 ^b^	55.7153 ^a^	0.1382	0.0001
Yellowness (b*) ^2^	21.2451 ^a^	20.7982 ^b^	19.9741 ^c^	20.0435 ^c^	0.1465	0.0001

^1^ Lightness (L*) values are a measure of lightness; a larger value indicates a lighter color. ^2^ Yellowness (b*) values are a measure of yellowness, a larger value indicates a more yellow color (−60 = blue, +60 = yellow). ^a,b,c^ Means lacking a common superscript letter differ (*p* ≤ 0.05). * SEM, Standard Error of the Mean.

**Table 3:** Main effect of day on instrumental fresh surface color of ground beef patties during a simulated retail display.

	Day of Display					
	0	3	5	7	SEM *	*p*-Value
Lightness (L*) ^1^	56.144 ^a^	54.367 ^b^	53.559 ^c^	53.559 ^c^	0.1382	0.0001
Yellowness (b*) ^2^	25.246 ^a^	21.205 ^b^	17.804 ^c^	17.804 ^c^	0.1465	0.0001

^1^ Lightness (L*) values are a measure of lightness; a larger value indicates a lighter color. ^2^ Yellowness (b*) values are a measure of yellowness, a larger value indicates a more yellow color (−60 = blue, +60 = yellow). ^a,b,c^ Means lacking a common superscript letter differ (*p* ≤ 0.05). * SEM, Standard Error of the Mean.

**Table 4:** Influence of oat protein inclusion main effects on lipid oxidation (TBARS) on fresh ground beef patties after a simulated retail display.

		**Treatment**				
	**0%**	**1.5%**	**3.5%**	**4.5%**	SEM *	***p*-Value**
TBARS ^1^	1.6941	1.7293	1.8354	1.8832	0.0789	0.2995
		**Day**				
	**0**	**3**	**5**	**7**	SEM *	***p*-Value**
TBARS ^1^	2.421 ^a^	1.531 ^b^	--	1.404 ^b^	0.0683	0.0001

^1^ Thiobarbituric acid reactive substances (TBARS) is a measure of lipid oxidation with a larger value indicating a greater amount of oxidation. Interactive treatment × day of lipid oxidation (*p* = 0.2314). ^a,b^ Means lacking a common superscript letter differ (*p* ≤ 0.05). * SEM, Standard Error of the Mean.

**Table 5:** Main effects of treatment on cooked ground beef patties containing varying percentages of oat protein on instrumental cooked color.

	Treatment					
	0%	1.5%	3.5%	4.5%	SEM *	*p*-Value
Lightness (L*) ^1^	55.3082	55.5011	55.4110	55.3773	0.1950	0.9165
Redness (a*) ^2^	17.1937 ^a^	15.8252 ^b^	15.3455 ^b^	14.4602 ^c^	0.2436	0.0001
Yellowness (b*) ^3^	16.3984	16.7297	16.5903	16.7103	0.1803	0.5507
Hue Angle ^4^	1.5525 ^c^	1.5543 ^b^	1.5546 ^b^	1.5557 ^a^	0.0002	0.0001
Chroma ^5^	23.7627 ^a^	23.0341 ^a,b^	22.6082 ^b,c^	22.1023 ^c^	0.2543	0.0004
Red to Brown ^6^	1.9873 ^a^	1.8161 ^b^	1.8018 ^b^	1.6704 ^c^	0.0448	0.0002

^1^ Lightness (L*) values are a measure of lightness; a larger value indicates a lighter color. ^2^ Redness (a*) values are a measure of redness, larger values indicate a redder color (−60 = green, +60 = red). ^3^ Yellowness (b*) values are a measure of yellowness, a larger value indicates a more yellow color (−60 = blue, +60 = yellow). ^4^ Hue angle represents the change from the true red axis with a greater angle indicating a greater shift from red to yellow (Hue angle = tan − 1 b*/a*). ^5^ Chroma is a measure of the total color (larger value indicates more total color). ^6^ Red to Brown is a ratio of 630:580 nm which represents a change in the color of red to brown (larger value indicates a more red color). ^a,b,c^ Means lacking a common superscript letter differ (*p* ≤ 0.05). * SEM, Standard Error of the Mean.

## Data Availability

Not applicable.
